# Which are the most sensitive search filters to identify randomized controlled trials in MEDLINE?

**DOI:** 10.5195/jmla.2020.912

**Published:** 2020-10-01

**Authors:** Julie Glanville, Eleanor Kotas, Robin Featherstone, Gordon Dooley

**Affiliations:** 1 julie@glanville.info, York Health Economics Consortium, University of York, York, United Kingdom; 2 eleanor.kotas@york.ac.uk, York Health Economics Consortium, University of York, York, United Kingdom; 3 rfeatherstone@cochrane.org, Editorial and Methods Department, Cochrane, London, United Kingdom; 4 Gordon@metaxis.com, Metaxis, Oxford, United Kingdom

## Abstract

**Objective:**

The Cochrane Handbook of Systematic Reviews contains search filters to find randomized controlled trials (RCTs) in Ovid MEDLINE: one maximizing sensitivity and another balancing sensitivity and precision. These filters were originally published in 1994 and were adapted and updated in 2008. To determine the performance of these filters, the authors tested them and thirty-six other MEDLINE filters against a large new gold standard set of relevant records.

**Methods:**

We identified a gold standard set of RCT reports published in 2016 from the Cochrane CENTRAL database of controlled clinical trials. We retrieved the records in Ovid MEDLINE and combined these with each RCT filter. We calculated their sensitivity, relative precision, and f-scores.

**Results:**

The gold standard comprised 27,617 records. MEDLINE searches were run on July 16, 2019. The most sensitive RCT filter was Duggan et al. (sensitivity=0.99). The Cochrane sensitivity-maximizing RCT filter had a sensitivity of 0.96 but was more precise than Duggan et al. (0.14 compared to 0.04 for Duggan). The most precise RCT filters had 0.97 relative precision and 0.83 sensitivity.

**Conclusions:**

The Cochrane Ovid MEDLINE sensitivity-maximizing RCT filter can continue to be used by Cochrane reviewers and to populate CENTRAL, as it has very high sensitivity and a slightly better precision relative to more sensitive filters. The results of this study, which used a very large gold standard to compare the performance of all known RCT filters, allows searchers to make better informed decisions about which filters to use for their work.

## INTRODUCTION

The effective retrieval of evidence is essential to achieve robust evidence synthesis, support health care decision making, and conduct health research. In particular, the efficient retrieval of evidence from randomized controlled trials (RCTs) is core to many systematic reviews [1–4] and essential to the systematic reviews produced by Cochrane (formerly, the Cochrane Collaboration).

Cochrane's study identification processes for trials in MEDLINE have been informed by RCT filters that were developed in 1994 and updated and adapted in 2008 [1]. Since their development, these Cochrane RCT filters have been widely used to search MEDLINE by reviewers who are internal and external to Cochrane [5]. The Cochrane RCT filters have also been used to populate the CENTRAL register of controlled trials. CENTRAL is built by collecting reports of RCTs from MEDLINE via PubMed, Embase, and a range of other resources [6]. CENTRAL is a primary resource for information specialists, librarians, and systematic reviewers seeking trial literature, as it is the largest database specifically collecting reports of RCTs in health care.

Over the last two decades, many authors have developed search filters to enable RCT retrieval when searching bibliographic databases [7]. The Cochrane RCT filters for MEDLINE have not been tested against a large set of known RCT records (i.e., gold standard) since 2008. To ensure that Cochrane is using a high performing strategy in terms of high sensitivity and that CENTRAL is populated by a sensitive MEDLINE strategy, Cochrane commissioned a performance test of the Cochrane RCT filters for MEDLINE alongside other published RCT filters.

## METHODS

### Identifying search filters

The Cochrane RCT filters for Ovid MEDLINE were identified from the Cochrane Handbook [1]. The authors identified other Ovid MEDLINE RCT filters from a review by McKibbon, Wilczynski, and Haynes [8] and the ISSG search filters resource [9]. The filters were run exactly as they were reported, which meant that some but not all of the filters had animal study exclusions as part of the filter. The filters are listed in supplemental Appendix A.

We used the Ovid interface because the Cochrane RCT filters were developed for this interface, and many comparator filters for this interface were available. Ovid also allowed the speedy preparation of gold standard sets of records and permitted us to store and rerun the filters easily.

### Developing the gold standard

Filter performance tests require as large a gold standard of relevant RCTs as possible. To the best of our knowledge, CENTRAL provides the largest available curated collection of reports of RCTs. We identified all records from CENTRAL that were published in 2016 and indexed in MEDLINE. That year was chosen to ensure the filters were being tested for the most recent year for which as many publications as possible would have been identified for inclusion in MEDLINE. We identified the PubMed identifiers (IDs) of these records and then searched for the identifiers in Ovid MEDLINE.

### Testing filter performance

In Ovid MEDLINE, each filter was combined with the gold standard set to determine how many gold standard records each filter would retrieve. Sensitivity, relative precision, and f-score were calculated for each filter (Table 1). Relative precision is a pragmatic measure allowing the comparison of filters in this study and reflects the fact that the records were published in a single year (i.e., 2016) and the assumption that CENTRAL records represent the gold standard set of reports of RCTs in MEDLINE.

**Table 1:** Definitions of sensitivity, relative precision, and f-score

Measure	Definition
Sensitivity	Number of relevant records (i.e., gold standard records) retrieved by a filter divided by the total number of relevant records.
Relative precision	Number of retrieved relevant records divided by the total number of records retrieved by a filter within a specific publication date range.
f-score	Average of sensitivity and relative precision values, used to measure the accuracy of a filter. An f-score of 1 reflects an ideal balance between sensitivity and relative precision [10].

## RESULTS

### Search filters

In addition to the two Cochrane RCT filters, we identified thirty-six other RCT filters that were all listed in a review by McKibbon, Wilczynski, and Haynes [8]. No further filters were identified from the ISSG search filters resource (searched on March 7, 2019).

### Gold standard

We identified 29,428 IDs for reports of RCTs that were indexed in MEDLINE and available in CENTRAL. Of these IDs, 27,617 yielded results in Ovid MEDLINE. The discrepancy between the total number of IDs and the number of IDs retrieved in MEDLINE was due to the presence of duplicate records with different identifiers in CENTRAL for single publications; however, these duplicates had been resolved in MEDLINE.

### Search filter performance

Table 2 shows the sensitivity, relative precision, and f-score of all 38 RCT filters, in order of sensitivity. Tables showing the same data in order of relative precision and f-score are in supplemental Appendix B. Eight filters offer sensitivity of 95% or higher, with relative precision ranging from 0.04 to 0.96.

**Table 2:** Sensitivity, relative precision, and f-score of 38 randomized controlled trials (RCTs) filters (ordered by sensitivity)

Rank	RCT filter number	Name of filter	Sensitivity	Relative precision	f-score
1	RCT filter 1	Duggan et al. (1997) [11]*	0.99	0.04	0.51
2	RCT filter 35	Dumbrigue et al. 7 (2000) [12]	0.98	0.04	0.51
3	RCT filter 4	Robinson and Dickersin 2 (2002) [13]	0.97	0.10	0.54
4	RCT filter 5	Cochrane D (2011) [1]	0.97	0.10	0.54
5	RCT filter 6	Miner Library Rochester strategy 1 (Miner 1) (not originally validated) (2009) [14]*	0.97	0.13	0.55
6	RCT filter 2	Robinson and Dickersin 1 (2002) [13]	0.97	0.10	0.53
7	RCT filter 3	Clinical Queries sensitive (2005) [15]*	0.97	0.12	0.54
8	Cochrane RCT filter 1	Sensitivity maximizing RCT filter	0.96	0.14	0.55
9	Cochrane RCT filter 2	Sensitivity and precision maximizing RCT filter	0.93	0.46	0.69
10	RCT filter 11	Marson and Chadwick comprehensive (Marson 1) (1996) [16]*	0.92	0.26	0.59
11	RCT filter 14	Adams et al. skilled (Adams et al. 2) (1994) [17]*	0.92	0.29	0.61
12	RCT filter 15	Chow 2 (1993) [18]*	0.91	0.35	0.63
13	RCT filter 16	Royle and Waugh 1 (2008) [19]*	0.91	0.35	0.63
14	RCT filter 17	Marson and Chadwick basic (Marson 2) (1996) [16]*	0.91	0.36	0.63
15	RCT filter 19	Clinical Queries balanced (2005) [15]*	0.89	0.53	0.71
16	RCT filter 8	Glanville and Lefebvre strategy A (2006) [20]*	0.88	0.11	0.49
17	RCT filter 18	Scottish Intercollegiate Guidelines Network (SIGN) (undated) (not originally validated) [21]*	0.87	0.39	0.63
18	RCT filter 21	Cochrane A (2011) [1] Dickersin et al. 1 (1994) [22]	0.87	0.92	0.89
19	RCT filter 20	Dumbrigue et al. 1 (2000) [12]*	0.87	0.23	0.55
20	RCT filter 23	Nwosu et al. (1998) [23]*	0.86	0.96	0.91
21	RCT filter 25	Corrao et al. (2006) [24]*	0.85	0.83	0.84
22	RCT filter 7	Glanville and Lefebvre strategy D (2006) [20]*	0.84	0.13	0.49
23	RCT filter 26	Clinical Queries specific (2005) [15]*	0.84	0.90	0.87
24	RCT filter 24	Dumbrigue et al. 3 (2000) [12]*	0.84	0.62	0.73
25	RCT filter 28	Chow 1 [18] Glanville and Lefebvre E (2006) [20] Royle and Waugh 2 (2007) [19] Dumbrigue et al. 9 (2000) [12]*	0.83	0.97	0.90
26	RCT filter 9	Glanville and Lefebvre strategy B (2006) [20]*	0.83	0.16	0.50
27	RCT filter 10	Cochrane B (2011) [1] Dickersin et al. 2 (1994) [22]	0.82	0.26	0.54
28	RCT filter 12	Miner Library Rochester strategy 2 (Miner 2 not originally validated) (2009) [14]*	0.81	0.30	0.56
29	RCT filter 27	Jadad and McQuay (1993) [25]*	0.80	0.27	0.54
30	RCT filter 29	Eisinga et al. (2007) [26]*	0.76	0.33	0.55
31	RCT filter 13	Glanville and Lefebvre strategy C (2006) [20]*	0.76	0.34	0.55
32	RCT filter 30	Cochrane C (2011) [1] Dickersin et al. 3 (1994) [22]	0.74	0.09	0.42
33	RCT filter 32	Dumbrigue et al. 6 (2000) [12]	0.64	0.60	0.62
34	RCT filter 31	Dumbrigue et al. 5 (2000) [12]*	0.59	0.13	0.36
35	RCT filter 33	Dumbrigue et al. 2 (2000) [12]*	0.41	0.36	0.39
36	RCT filter 34	Dumbrigue et al. 4 (2000) [12]*	0.21	0.55	0.38
37	RCT filter 36	Adams et al. standard (Adams et al. 1) (1994) [17]*	0.03	0.06	0.05
38	RCT filter 22	Glanville and Lefebvre strategy F (2006) [20] Dumbrique 8 (2000) [12]*	0.02	0.07	0.04

Note: Shaded rows are the Cochrane filters.

* The filter excludes animal studies.

As shown in Table 2, the most sensitive filter is Duggan et al. [11]. However, both of the Cochrane RCT [1] filters continue to perform well in this large gold standard of records published in 2016, despite being developed more than 10 years ago. The Cochrane RCT [1] filters rank eighth and ninth with sensitivities of 0.96 and 0.93, respectively. Despite slightly lower sensitivity, the Cochrane [1] filters are more precise (0.14 and 0.46, respectively) than any of the filters that rank above them in terms of sensitivity. Comparing the most sensitive filters with the Cochrane RCT filters [1], the Duggan et al. [11] filter does not remove animal studies or limit to human studies, whereas the second most sensitive filter (Dumbrigue et al. 7 [12]) and the Cochrane RCT [1] filters do. We would expect the Dumbrigue et al. [12] and Cochrane RCT filters [1] to be more relatively precise since they have animal study exclusions. Three of the 4 filters (Duggan et al. [11] and Cochrane RCT filters [1]) include “randomized controlled trial.pt,” and all 4 of the filters include “trial,” the word “random” in some variation (i.e., random or randomly) and either “clinical trial” or “trial.” Other than having these terms in common, these filters are not very comparable.

**Figure 1:**
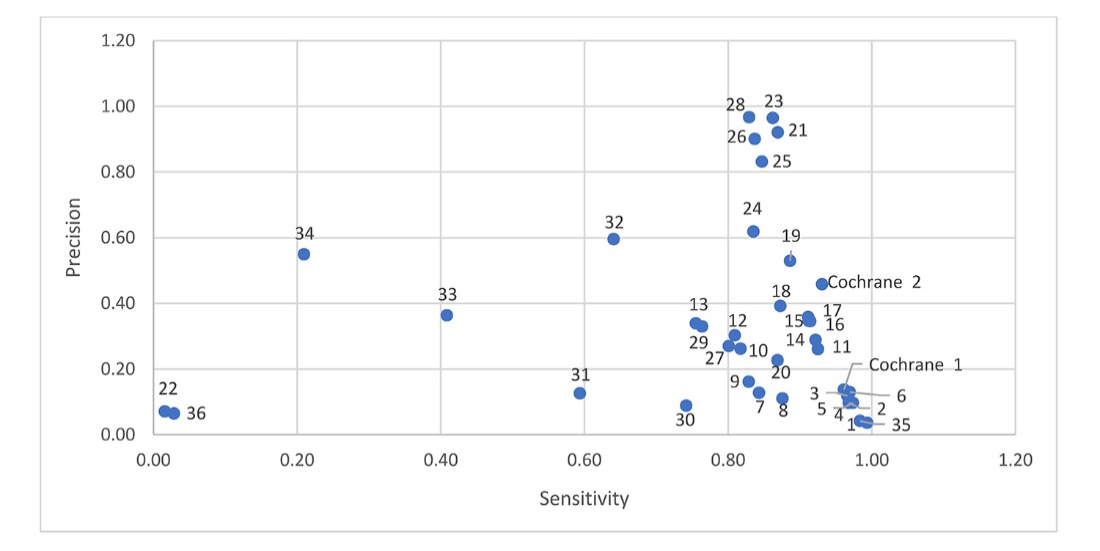
Sensitivity and relative precision of 38 randomized controlled trial (RCT) filters

Note: The numbers next to the data points are associated with the RCT filters in Table 2.

Figure 1 shows a scatter plot of sensitivity and relative precision. Four filters offer reasonable sensitivity (0.83) with higher relative precision (0.97) [8, 12, 19, 20]. The Cochrane sensitivity and precision maximizing RCT filter ranks tenth, with better sensitivity than the other filters above it, but with lower relative precision (0.46). Comparing the most relatively precise filters (Chow 1 [18], Dumbrigue et al. 9 [12], Royle and Waugh 2 [19], Glanville and Lefebvre E [20], and Nwosu et al. [23]) to the Cochrane RCT filters, all of these filters include “randomized controlled trial.pt,” and only the Cochrane RCT filters exclude animal studies. We would expect the Cochrane RCT filters to be more relatively precise since they exclude animal studies. The Chow 1 [18], Dumbrigue et al. 9 [12], Royle and Waugh 2 [19], Glanville and Lefebvre E [20], and Nwosu et al. [23] filters are short filters, containing 1 and 2 lines respectively. There are few similarities between these filters.

Assessing the RCT filters by f-score demonstrates that filters are subject to a trade-off between sensitivity and relative precision. If a RCT filter with a balance of sensitivity and precision is required, the f-score suggests using the Nwosu et al. [23] RCT filter (f-score of 0.91).

## DISCUSSION

We have provided an up-to-date analysis of the performance of RCT search filters in Ovid MEDLINE using a very large gold standard set of relevant records. We were able to compile a very large gold standard set of records without needing to rely on relative recall approaches. The relative recall approach is widely used [27] to generate gold standards (as well as hand searching techniques) for filter design and to investigate search performance. Relative recall uses published systematic reviews to generate a gold standard and is a useful and economical way to achieve these, but this approach is highly reliant on the quality of the searches undertaken to populate the reviews. CENTRAL provides a highly valuable source of RCT records that can be used to create gold standards.

The consistency of the performance of the Cochrane filters over time is sustained, as is the performance of many other filters. The last time the filters were analyzed and adapted in 2008 [1], the Cochrane sensitivity maximizing filter had a sensitivity ranking of 0.99 compared with the ranking of 0.96 in the present analysis, suggesting that the terminology used to describe RCTs has not changed substantially over time. This does not mean that terminology would not change in the future, but there is a suggestion that the terminology is currently stable, and reassessment of filter performance once a decade may be acceptable. However, with the continued development of machine learning and language classifiers [28], the use of search filters in the context of systematic reviews may become ever more sensitive and less precise, because the screening can be done economically with a machine classifier.

Filters do miss relevant studies. When we looked at the 1,098 gold standard records that were not retrieved by the Cochrane RCT filter, we could see that 708 did not contain any of the terms in the RCT filter and so would not have been retrieved, suggesting that they were identified and added to CENTRAL using other identification routes such as hand searching. Ninety-nine records were animal studies and so would have been removed by the animal studies line in the RCT filter, if it had been used. Sixty-one records are now in PubMed with a date that is different from 2016, reflecting that database records change more than one might expect; for example, there may be changes to many fields over time, including dates, page numbers, and indexing terms. The remaining 230 results contained variations of the words in the search filter; for example, a record may have “subgroup” or “trials,” whereas the search terms in the RCT filter are “groups” and “trials,” respectively.

Searchers may also want to incorporate any indexing changes that have occurred since the filter was developed. The RCT search filter strategies were tested as originally published, because they were usually the results of research efforts and changes to them would not be possible to validate using their original gold standards. However, we carried out an exploratory test with the Cochrane RCT filters to explode the Medical Subject Headings (MeSH) “randomized controlled trial/” and to adjust “randomized.ab” to “randomized.ti,ab,” and we found that sensitivity, relative precision, and f-score remained unchanged. We note that such explorations are interesting and reassuring, but searchers who are interested in filter development or improvement should ideally undertake these activities in a structured way and validate the filters. Filter development is grounded in an awareness that increasing sensitivity (e.g., through use of more truncation) nearly always impacts relative precision.

We used CENTRAL as the source of our gold standard, as this database is designed to contain only reports of controlled clinical trials. As such, we acknowledge that some of the records in the gold standard may not be reports of controlled clinical trials due to indexing errors or misclassification. However, the proportion of such records is likely to be small and the gold standard is very large, so the impact is likely to be minor.

CENTRAL is partly compiled by using the Cochrane RCT filters for MEDLINE, so it could be argued that Cochrane filters perform better than other filters because they were used, in part, to generate the records. This may be partly true, but many of the MEDLINE records in CENTRAL were added from a range of other routes and methods, so any advantage is likely to be diluted. Of the 29,428 gold standard records retrieved from CENTRAL, 5,619 of these records were not sourced using the Cochrane RCT filter. This means that 19% of MEDLINE records in CENTRAL were identified by means other than the Cochrane RCT filter. The Cochrane RCT filter also performs well in identifying those records that have been added to CENTRAL from other routes such as hand searching or by reviewers assessing the full text of records.

The filters that we used were run in MEDLINE as they were reported by filter authors. Some, but not all, of these filters excluded animal studies. We might expect filters that excluded animal studies to have slightly better relative precision than filters that did not. However, we see that although a large proportion of filters that excluded animal studies were at the higher end of the relative precision ranking, there were also filters that excluded animal studies that ranked very low in terms of relative precision.

Because the search was conducted on records with 2019 indexing, we do not know how the filters would have actually fared in 2016, when some records might have had different or no indexing. Therefore, this analysis showed how filters perform in records with a date of 2016 and current indexing in 2019.

Relative precision is a pragmatic measure reflecting the use of a gold standard set of records published in 2016 and the assumption that the CENTRAL records represent a gold standard set of reports of RCTs in MEDLINE. As noted above, MEDLINE records are identified for inclusion in CENTRAL via various routes, which enhances its claim to be a gold standard. However, it is likely that there still remain reports of RCTs in MEDLINE in 2016 that have yet to be identified as such and are not yet included in CENTRAL. In that case, each filter's sensitivity could be slightly higher or lower than the results presented here.

The Cochrane Ovid MEDLINE sensitivity maximizing RCT filter can continue to be used by Cochrane reviewers and to populate CENTRAL, as it has very high sensitivity and a slightly better relative precision than the more sensitive filters. With the added value of this large-scale study comparing the performance of all known RCT filters, searchers can now make more informed decisions about which filters to use for their work.

## Data Availability

The data associated with this paper can be found at 10.5281/zenodo.3625063.

## SUPPORTING AGENCY

Cochrane.

## SUPPLEMENTAL FILES

**Appendix A:** Cochrane Highly Sensitive Search Strategies, Ovid format, and randomized controlled trial filters

**Appendix B:** Tables, ordered by precision and f-score
