# Supplementary material for: Which are the most sensitive search filters to identify randomized controlled trials in MEDLINE?
Source: J Med Libr Assoc. 2020 Oct 1;108(4):556–63. doi: 10.5195/jmla.2020.912 (PMC7524635; doi:10.5195/jmla.2020.912)
Supplement: Supplementary file 1 — Appendix A: Cochrane Highly Sensitive Search Strategies, Ovid format, and randomized controlled trial filters [file jmla-108-4-556-s01.pdf]

JMLA Journal of the Medical Library Association

SC
Supplemental content to
J Med Libr Assoc. Oct;108(4):dx.doi.org/10.5195/jmla.2020.912
www.jmla.mlanet.org
© Glanville, Kotas, Featherstone, Dooley 2020

Which are the most sensitive search filters to identify randomized controlled trials in MEDLINE?

Julie Glanville; Eleanor Kotas; Robin Featherstone; Gordon Dooley

APPENDIX A

Cochrane Highly Sensitive Search Strategies, Ovid format, and randomized controlled trial filters

Cochrane Highly Sensitive Search Strategy for identifying randomized trials in MEDLINE: sensitivity- and precision-maximizing version (2008 revision); Ovid format
Source: The Cochrane Handbook, section 6.4.d, search filters

1	randomized controlled trial.pt.
2	controlled clinical trial.pt.
3	randomized.ab.
4	placebo.ab.
5	drug therapy.fs.
6	randomly.ab.
7	trial.ab.
8	groups.ab.
9	1 or 2 or 3 or 4 or 5 or 6 or 7 or 8
10	exp animals/ not humans.sh.
11	9 not 10

Cochrane Highly Sensitive Search Strategy for identifying randomized trials in MEDLINE: sensitivity- and precision-maximizing version (2008 revision); Ovid format
Source: The Cochrane Handbook, section 6.4.d, search filters

1	randomized controlled trial.pt.
2	controlled clinical trial.pt.
3	randomized.ab.
4	placebo.ab.
5	clinical trials as topic.sh.
6	randomly.ab.
7	trial.ti.
8	1 or 2 or 3 or 4 or 5 or 6 or 7
9	exp animals/ not humans.sh.
10	8 not 9

JMLA Journal of the Medical Library Association

SC
Supplemental content to
J Med Libr Assoc. Oct;108(4):dx.doi.org/10.5195/jmla.2020.912
www.jmla.mlanet.org
© Glanville, Kotas, Featherstone, Dooley 2020

The thirty-six randomized controlled trial filters taken from McKibbon KA, Wilczynski NL, Haynes RB. Retrieving randomized controlled trials from MEDLINE: a comparison of 38 published search filters. Health Inf Libr J. 2009 Sep;26(3):187–202.

These are in highest to lowest sensitivity according to the paper.

The Cochrane search strategies are not included in this table so there are thirty-six, not thirty-eight.

	Filter name	Filter strategy
1	Duggan et al. (1997) [11]	1 effect:.tw.<br>2 trial:.tw.<br>3 investigat:.tw.<br>4 random:.tw.<br>5 control:.tw.<br>6 experimental.tw.<br>7 double blind.tw.<br>8 compar:.tw.<br>9 matched.tw.<br>10 blind.tw.<br>11 examine:.tw.<br>12 study.tw.<br>13 comparative study.sh.<br>14 randomized controlled trial.pt.<br>15 OR/1–14
2	Robinson and Dickersin 1 (2002) [13]	1 randomized controlled trial.pt.<br>2 clinical trials as topic.sh. (updated MeSH heading 2007)<br>3 randomized controlled<br>4 random allocation.sh.trials as topic.sh.<br>5 double-blind method.sh.<br>6 single-blind method.sh.<br>7 clinical trial.pt.<br>8 explode clinical trials as topic<br>9 (clinic: adj25 trial:).ti,ab.<br>10 ((singl: or doubl: or trebl: or tripl:) adj25<br>(blind: or mask:)).ti,ab.<br>11 placebos.sh.<br>12 placebo:.ti,ab.<br>13 random:.ti,ab.<br>14 research design.sh.<br>15 comparative study.sh.<br>16 explode evaluation studies

SC
Supplemental content to
J Med Libr Assoc. Oct;108(4):dx.doi.org/10.5195/jmla.2020.912
www.jmla.mlanet.org
© Glanville, Kotas, Featherstone, Dooley 2020

	Filter name	Filter strategy
		17 follow-up studies.sh.<br>18 prospective studies.sh.<br>19 (control: or prospectiv: or volunteer:).ti,ab.<br>20 OR/1–19<br>21 animals.sh. NOT humans.sh<br>22 20 NOT 21
3	Clinical Queries sensitive (2005) [15]	1 clinical trial.mp.<br>2 clinical trial.pt.<br>3 random:.mp.<br>4 tu.xs.<br>5 OR/1–4
4	Robinson and Dickersin 2 (2002) [13]	1 randomized controlled trial.pt.<br>2 controlled clinical trial.pt.<br>3 clinical trials as topic.sh. (updated Medical Subject Headings [MeSH] term 2007)<br>4 random allocation.sh.<br>5 double-blind method.sh.<br>6 single-blind method.sh.<br>7 clinical trial.pt.<br>8 explode clinical trials as topic<br>9 (clinic: adj25 trial:).ti,ab.<br>10 ((singl: or doubl: or trebl: or tripl:) adj25 (blind: or mask:)).ti,ab.<br>11 placebos.sh.<br>12 placebo:.ti,ab.<br>13 random:.ti,ab.<br>14 research design.sh.<br>15 comparative study.sh.<br>16 explode evaluation studies<br>17 follow-up studies.sh.<br>18 prospective studies.sh.<br>19 (control: or prospectiv: or volunteer:).ti,ab.<br>20 cross-over studies.sh.<br>21 latin square:.tw.<br>22 OR/1–21<br>23 animals.sh. NOT humans.sh<br>24 22 NOT 23

SC
Supplemental content to
J Med Libr Assoc. Oct;108(4):dx.doi.org/10.5195/jmla.2020.912
www.jmla.mlanet.org
© Glanville, Kotas, Featherstone, Dooley 2020

	Filter name	Filter strategy
5	Cochrane D (2011) [1]	1 randomized controlled trial.pt.<br>2 controlled clinical trial.pt.<br>3 randomized controlled trials as topic.sh.<br>4 random allocation.sh.<br>5 double-blind method.sh.<br>6 single-blind method.sh.<br>7 clinical trial.pt.<br>8 explode clinical trials as topic (updated MeSH term 2007)<br>9 (clin: adj25 trial:).ti, ab.<br>10 (single: OR doubl: OR trebl: OR tripl:) adj25 (blind: OR mask:).ti,ab.<br>11 placebos.sh.<br>12 placebo:.ti,ab.<br>13 random:.ti,ab.<br>14 research design.sh.<br>15 comparative study.sh.<br>16 explode evaluation studies<br>17 follow up studies.sh.<br>18 prospective studies.sh.<br>19 (control: OR prospective: OR volunteer:).ti,ab.<br>20 OR/1–19<br>21 animals.sh. NOT humans.sh<br>22 20 NOT 21
6	Miner Library Rochester strategy 1 (Miner 1) (not originally validated) (2009) [14]	1 explode research design<br>2 explode clinical trials as topic<br>3 comparative study.sh. or placebos.sh.<br>4 explode treatment outcome<br>5 double-blind method.sh. or single-blind method.sh.<br>6 ((single or double or triple) adj blind:3).ti,ab.<br>7 random:.ti,ab.<br>8 controlled clinical trial.pt.<br>9 randomized controlled trial.pt.<br>10 practice guideline.pt.<br>11 clinical trial.pt.<br>12 (clinical adj trial:1).ti,ab.<br>13 placebo:1.ti,ab.<br>14 clinical protocols.sh. or feasibility studies.sh. or pilot projects.sh.<br>15 explode epidemiologic research design

SC
Supplemental content to
J Med Libr Assoc. Oct;108(4):dx.doi.org/10.5195/jmla.2020.912
www.jmla.mlanet.org
© Glanville, Kotas, Featherstone, Dooley 2020

	Filter name	Filter strategy
		16 (control:3 adj trial:1).ti,ab.<br>17 OR/1–16
7	Glanville and Lefebvre strategy D (2006) [20]	1 clinical trial.pt.<br>2 randomized.ab.<br>3 placebo.ab.<br>4 clinical trials as topic.sh. (updated MeSH term 2007)<br>5 randomly.ab.<br>6 trial.ti.<br>7 drug therapy.fs.<br>8 effects.ti.<br>9 comparative study.sh.<br>10 OR/1–9
8	Glanville and Lefebvre strategy A (2006) [20]	1 clinical trial.pt.<br>2 randomized.ab.<br>3 placebo.ab.<br>4 drug therapy.fs.<br>5 randomly.ab.<br>6 trial.ab.<br>7 groups.ab.<br>8 OR/1–7
9	Glanville and Lefebvre strategy B (2006) [20]	1 clinical trial.pt.<br>2 randomized.ab.<br>3 placebo.ab.<br>4 drug therapy.fs.<br>5 randomly.ab.<br>6 trial.ab.<br>7 OR/1–6
10	Cochrane B (2011) [1]*<br>Dickersin et al. 2 (1994) [22]*	1 clinical trial.pt.<br>2 explode clinical trials as topic<br>3 (clin: adj25 trial:).ti, ab.<br>4 (single: OR doubl: OR trebl: OR tripl:)<br>adj25 (blind: OR mask:).ti,ab.<br>5 placebos.sh.<br>6 placebo:.ti,ab.<br>7 random:.ti,ab.<br>8 rese arch design.sh.<br>9 OR/1–8<br>10 animals.sh. NOT humans.sh<br>11 9 NOT 10

SC
Supplemental content to
J Med Libr Assoc. Oct;108(4):dx.doi.org/10.5195/jmla.2020.912
www.jmla.mlanet.org
© Glanville, Kotas, Featherstone, Dooley 2020

	Filter name	Filter strategy
11	Marson and Chadwick comprehensive (Marson 1) (1996) [16]	1 randomized controlled trial.pt.<br>2 randomized controlled trials as topic.sh.<br>3 double-blind method.sh.<br>4 single-blind method.sh.<br>5 clinical trial.pt.<br>6 exp clinical trials as topic<br>7 clinic: trial:.tw.<br>8 singl: blind:.tw. or singl: mask:.tw.<br>9 doubl: blind:.tw.<br>10 doubl: mask:.tw.<br>11 tripl: blind.tw.<br>12 tripl: mask:.tw.<br>13 placebos.sh.<br>14 random:.tw.<br>15 explode research design<br>16 OR/1–15
12	Miner Library Rochester strategy 2 (Miner 2 not originally validated) (2009) [14]	1 double-blind method.sh. or single-blind method.sh.<br>2 random:.ti,ab.<br>3 practice guideline.pt.<br>4 clinical trial.pt.<br>5 placebo:1.ti,ab.<br>6 OR/1–5
13	Glanville and Lefebvre strategy C (2006) [20]	1 clinical trial.pt.<br><br>2 randomized.ab.<br>3 placebo.ab.<br>4 clinical trials as topic.sh.<br>(updated MeSH heading 2007)<br>5 randomly.ab.<br>6 trial.ti.<br>7 OR/1–6
14	Adams et al. skilled (Adams 2) (1994) [17]	1 exp clinical trials as topic<br>2 clinical: trial:.tw.<br>3 random:.tw.<br>4 random allocation.sh.<br>5 clinical trials as topic.sh. (updated MeSH term 2007)<br>6 randomized controlled trial.pt.<br>7 singl: blind:.tw.

© Glanville, Kotas, Featherstone, Dooley 2020

	Filter name	Filter strategy
		8 single-blind method.sh.<br>9 doubl: blind:.tw.<br>10 double-blind method.sh.<br>11 tripl: blind:.tw.<br>12 trebl: blind:.tw.<br>13 doubl: mask:.tw.<br>14 OR/1–13
15	Chow 2 (1993) [18]	1 (double-blind: or random:).af.
16	Royle and Waugh 1 (2008) [19]	1 random:.af.
17	Marson and Chadwick basic (Marson 2) (1996) [16]	1 randomized controlled trial.pt.<br>2 randomized controlled trials as topic.sh.<br>3 random:.tw.<br>4 OR/1–3
18	Scottish Intercollegiate Guidelines Network (SIGN) (undated) (not originally validated) [21]	1 randomized controlled trials as topic.sh.<br>2 randomized controlled trial.pt.<br>3 random allocation.sh.<br>4 double blind method.sh.<br>5 single blind method.sh.<br>6 clinical trial.pt.<br>7 explode clinical trials as topic<br>8 OR/1–7<br>9 (clinic: adj trial:1).tw.<br>10 ((singl: or doubl: or treb: or tripl:) adj (blind:3 or mask:3)).tw<br>11 placebos.sh.<br>12 placebo:.tw.<br>13 randomly allocated.tw.<br>14 (allocated adj2 random).tw.<br>15 OR/9–14<br>16 8 OR 15<br>17 case report. tw.<br>18 letter.pt.<br>19 historical article.pt.<br>20 review of reported cases.pt<br>21 review, multicase.pt.<br>22 OR/17–21<br>23 16 NOT 22

SC
Supplemental content to
J Med Libr Assoc. Oct;108(4):dx.doi.org/10.5195/jmla.2020.912
www.jmla.mlanet.org
© Glanville, Kotas, Featherstone, Dooley 2020

	Filter name	Filter strategy
19	Clinical Queries balanced (2005) [15]	1 randomized controlled trial.pt.<br>2 randomized.mp.<br>3 placebo.mp.<br>4 1 OR 2 OR 3
20	Dumbrigue et al. 1 (2000) [12]	1 randomized controlled trial.pt.<br>2 therapeutic use.fs.<br>3 random.tw.<br>4 OR/1–3
21	Cochrane A (2011) [1]*<br>Dickersin et al. 1 (1994) [22]*	1 randomized controlled trial.pt.<br>2 controlled clinical trial.pt.<br>3 controlled clinical trials as topic.sh. (previously: Randomized controlled trials.sh.)<br>4 random allocation.sh.<br>5 double blind method.sh.<br>6 single-blind method.sh.<br>7 OR/1–6<br>8 animals.sh. NOT humans.sh<br>9 7 NOT 8
22	Glanville and Lefebvre strategy F (2006) [20]<br>Dumbrigue et al. 8 (2000) [12]*	1 clinical trial.pt.
23	Nwosu et al. (1998) [23]	1 controlled clinical trial.pt.<br>2 randomized controlled trial.pt.<br>3 1 OR 2
24	Dumbrigue et al. 3 (2000) [12]	1 randomized controlled trial.pt.<br>2 random.tw.<br>3 OR/1–2
25	Corrao et al. (2006) [24]	1 randomized controlled trial.pt.<br>2 randomized controlled trial.mp.<br>3 randomized controlled trial.mp.<br>4 OR/1–3
26	Clinical Queries specific (2005) [15]	1 randomized controlled trial.mp.<br>2 randomized controlled trial.pt.<br>3 1 OR 2
27	Jadad and McQuay (1993) [25]	1 clinical trials.tw.<br>2 explode clinical trials as topic (updated MeSH term 2007)<br>3 random:.tw.<br>4 random allocation.sh.

JMLA Journal of the Medical Library Association

SC
Supplemental content to
J Med Libr Assoc. Oct;108(4):dx.doi.org/10.5195/jmla.2020.912
www.jmla.mlanet.org
© Glanville, Kotas, Featherstone, Dooley 2020

	Filter name	Filter strategy
		5 randomized controlled trials as topic.sh. (updated MeSH term 2007)<br>6 double-blind.tw.<br>7 double-blind method.sh.<br>8 OR/1–7
28	Chow 1 (1993) [18]*<br>Glanville and Lefebvre E (2006) [20]*<br>Royle and Waugh 2 (2008) [19]*<br>Dumbrigue et al. 9 (2000) [12]*	1 randomized controlled trial.pt.
29	Eisinga et al. (2005) [26]	1 random:.tw.
30	Cochrane C (2011) [1]*<br>Dickersin et al. 3 (1994) [22]*	1 comparative study.sh.<br>2 explode evaluation studies<br>3 follow up studies.sh.<br>4 prospective studies.sh.<br>5 (control: OR prospective: OR volunteer:).ti,ab.<br>6 OR/1–5<br>7 animals.sh. NOT humans.sh<br>8 6 NOT 7
31	Dumbrigue et al. 5 (2000) [12]	1 random allocation.sh.<br>2 comparative study.sh.<br>3 drug therapy.fs. OR placebo.tw.<br>4 controlled trial.tw.<br>5 OR/1–4
32	Dumbrigue et al. 6 (2000) [12]	1 humans.sh.<br>2 animals.sh.<br>3 randomized.tw.<br>4 randomized.tw.<br>5 3 OR 4<br>6 1 AND 5<br>7 6 NOT 2
33	Dumbrigue et al. 2 (2000) [12]	1 random.tw.<br>2 placebo.tw.<br>3 double.tw. and blind.tw.<br>4 controlled trial.tw.<br>5 OR/1–4
34	Dumbrigue et al. 4 (2000) [12]	1 placebo.tw.<br>2 double blind.tw.<br>3 OR/1–2

JMLA Journal of the Medical Library Association

SC
Supplemental content to
J Med Libr Assoc. Oct;108(4):dx.doi.org/10.5195/jmla.2020.912
www.jmla.mlanet.org
© Glanville, Kotas, Featherstone, Dooley 2020

	Filter name	Filter strategy
35	Dumbrigue et al. 7 (2000) [12]	1 humans.sh.<br>2 comparative study.sh.<br>3 clinical trial.pt.<br>4 AND/1–3
36	Adams et al. standard (Adams 1) (1994) [17]	1 explode clinical trials as topic<br>(updated MeSH heading 2007)

* Where there is more than one reference in a row, it means the filters were the same but referenced in different papers.
