# Supplementary material for: Which are the most sensitive search filters to identify randomized controlled trials in MEDLINE?
Source: J Med Libr Assoc. 2020 Oct 1;108(4):556–63. doi: 10.5195/jmla.2020.912 (PMC7524635; doi:10.5195/jmla.2020.912)
Supplement: Supplementary file 2 — Appendix B: Tables, ordered by precision and f-score [file jmla-108-4-556-s02.pdf]

JMLA Journal of the Medical Library Association

SC
Supplemental content to
J Med Libr Assoc. Oct;108(4):dx.doi.org/10.5195/jmla.2020.912
www.jmla.mlanet.org
© Glanville, Kotas, Featherstone, Dooley 2020

Which are the most sensitive search filters to identify randomized controlled trials in MEDLINE?

Julie Glanville; Eleanor Kotas; Robin Featherstone; Gordon Dooley

APPENDIX B

Tables, ordered by precision and f-score

Table B.1: Sensitivity, relative precision, and f-score of 38 RCT filters (ordered by precision and with filters with animal exclusions shaded green)

Rank	RCT filter number	Name of filter	Sensitivity	Relative precision	f-score
1	RCT filter 28	Chow 1 (1993) [18]<br>Glanville and Lefebvre E (2006) [20]<br>Royle and Waugh 2 (2007) [19]<br>Dumbrigue et al. 9 (2000) [12]	0.83	0.97	0.90
2	RCT filter 23	Nwosu et al. (1998) [23]	0.86	0.96	0.91
3	RCT filter 21	Cochrane A (2011) [1]<br>Dickersin et al. 1 (1994) [22]	0.87	0.92	0.89
4	RCT filter 26	Clinical Queries specific (2005) [15]	0.84	0.90	0.87
5	RCT filter 25	Corrao et al. (2006) [24]	0.85	0.83	0.84
6	RCT filter 24	Dumbrigue et al. 3 (2000) [12]	0.84	0.62	0.73
7	RCT filter 32	Dumbrigue et al. 6 (2000) [12]	0.64	0.60	0.62
8	RCT filter 34	Dumbrigue et al. 4 (2000) [12]	0.21	0.55	0.38
9	RCT filter 19	Clinical Queries balanced (2005) [15]	0.89	0.53	0.71
10	Cochrane RCT filter 2	Sensitivity and precision maximizing RCT filter	0.93	0.46	0.69
11	RCT filter 18	SIGN (Scottish Intercollegiate Guidelines Network) (undated) (not originally validated) [21]	0.87	0.39	0.63
12	RCT filter 33	Dumbrigue et al. 2 (2000) [12]	0.41	0.36	0.39
13	RCT filter 17	Marson and Chadwick basic (Marson 2) (1996) [16]	0.91	0.36	0.63
14	RCT filter 16	Royle and Waugh 1 (2008) [19]	0.91	0.35	0.63
15	RCT filter 15	Chow 2 (1993) [18]	0.91	0.35	0.63
16	RCT filter 13	Glanville and Lefebvre strategy C (2006) [20]	0.76	0.34	0.55
17	RCT filter 29	Eisinga et al. (2007) [26]	0.76	0.33	0.55

SC
Supplemental content to
J Med Libr Assoc. Oct;108(4):dx.doi.org/10.5195/jmla.2020.912
www.jmla.mlanet.org
© Glanville, Kotas, Featherstone, Dooley 2020

Rank	RCT filter number	Name of filter	Sensitivity	Relative precision	f-score
18	RCT filter 12	Miner Library Rochester strategy 2 (Miner 2) (not originally validated) (2009) [14]	0.81	0.30	0.56
19	RCT filter 14	Adams et al. skilled (Adams 2) (1994) [17]	0.92	0.29	0.61
20	RCT filter 27	Jadad and McQuay (1993) [25]	0.80	0.27	0.54
21	RCT filter 10	Cochrane B (2011) [1]<br>Dickersin et al. 2 (1994) [22]	0.82	0.26	0.54
22	RCT filter 11	Marson and Chadwick comprehensive (Marson 1) (1996) [16]	0.92	0.26	0.59
23	RCT filter 20	Dumbrigue et al. 1 (2000) [12]	0.87	0.23	0.55
24	RCT filter 9	Glanville and Lefebvre strategy B (2006) [20]	0.83	0.16	0.50
25	Cochrane RCT filter 1	Sensitivity maximizing RCT filter	0.96	0.14	0.55
26	RCT filter 6	Miner Library Rochester strategy 1 (Miner 1) (not originally validated) (2009) [14]	0.97	0.13	0.55
27	RCT filter 7	Glanville and Lefebvre strategy D (2006) [20]	0.84	0.13	0.49
28	RCT filter 31	Dumbrigue et al. 5 (2000) [12]	0.59	0.13	0.36
29	RCT filter 3	Clinical Queries sensitive (2005) [15]	0.97	0.12	0.54
30	RCT filter 8	Glanville and Lefebvre strategy A (2006) [20]	0.88	0.11	0.49
31	RCT filter 5	Cochrane D (2011) [1]	0.97	0.10	0.54
32	RCT filter 4	Robinson and Dickersin et al. 2 (2002) [13]	0.97	0.10	0.54
33	RCT filter 2	Robinson and Dickersin et al. 1 (2002) [13]	0.97	0.10	0.53
34	RCT filter 30	Cochrane C (2011) [1]<br>Dickersin et al. 3 (1994) [22]	0.74	0.09	0.42
35	RCT filter 22	Glanville and Lefebvre strategy F (2006) [9]<br>Dumbrigue et al. 8 (2000) [12]	0.02	0.07	0.04
36	RCT filter 36	Adams et al. standard (Adams et al. 1) (1994) [17]	0.03	0.06	0.05
37	RCT filter 35	Dumbrigue et al. 7 (2000) [12]	0.98	0.04	0.51
38	RCT filter 1	Duggan et al. (1997) [11]	0.99	0.04	0.51

SC
Supplemental content to
J Med Libr Assoc. Oct;108(4):dx.doi.org/10.5195/jmla.2020.912
www.jmla.mlanet.org
© Glanville, Kotas, Featherstone, Dooley 2020

Table B.2: Sensitivity, relative precision, and f-score of 38 RCT filters (ordered by f-score)

Rank	RCT filter number	Name of filter	Sensitivity	Relative precision	f-score
1	RCT filter 23	Nwosu et al. (1998) [23]	0.86	0.96	0.91
2	RCT filter 28	Chow 1 (1993) [18]<br>Glanville and Lefebvre E (2006) [20]<br>Royle and Waugh 2 (2008) [19]<br>Dumbrigue et al. 9 (2000) [12]	0.83	0.97	0.90
3	RCT filter 21	Cochrane A (2011) [1]<br>Dickersin et al. 1 (1994) [22]	0.87	0.92	0.89
4	RCT filter 26	Clinical Queries specific (2005) [15]	0.84	0.90	0.87
5	RCT filter 25	Corrao et al. (2006) [24]	0.85	0.83	0.84
6	RCT filter 24	Dumbrigue et al. 3 (2000) [12]	0.84	0.62	0.73
7	RCT filter 19	Clinical Queries balanced (2005) [15]	0.89	0.53	0.71
8	Cochrane RCT filter 2	Sensitivity and precision maximizing RCT filter	0.93	0.46	0.69
9	RCT filter 17	Marson and Chadwick basic (Marson 2) (1996) [16]	0.91	0.36	0.63
10	RCT filter 18	SIGN (Scottish Intercollegiate Guidelines Network) (undated) (not originally validated) [21]	0.87	0.39	0.63
11	RCT filter 15	Chow 2 (1993) [18]	0.91	0.35	0.63
12	RCT filter 16	Royle and Waugh 1 (2008) [19]	0.91	0.35	0.63
13	RCT filter 32	Dumbrigue et al. 6 (2000) [12]	0.64	0.60	0.62
14	RCT filter 14	Adams et al. skilled (Adams 2) (1994) [17]	0.92	0.29	0.61
15	RCT filter 11	Marson and Chadwick comprehensive (Marson 1) (1996) [16]	0.92	0.26	0.59
16	RCT filter 12	Miner Library Rochester strategy 2 (Miner 2) (not originally validated) (2009) [14]	0.81	0.30	0.56
17	RCT filter 6	Miner Library Rochester strategy 1 (Miner 1) (not originally validated) (2009) [14]	0.97	0.13	0.55
18	Cochrane RCT filter 1	Sensitivity maximizing RCT filter	0.96	0.14	0.55
19	RCT filter 20	Dumbrigue et al. 1 (2000) [12]	0.87	0.23	0.55
20	RCT filter 13	Glanville and Lefebvre strategy C (2006) [20]	0.76	0.34	0.55
21	RCT filter 29	Eisinga et al. (2007) [26]	0.76	0.33	0.55

SC
Supplemental content to
J Med Libr Assoc. Oct;108(4):dx.doi.org/10.5195/jmla.2020.912
www.jmla.mlanet.org
© Glanville, Kotas, Featherstone, Dooley 2020

Rank	RCT filter number	Name of filter	Sensitivity	Relative precision	f-score
22	RCT filter 3	Clinical Queries sensitive (2005) [15]	0.97	0.12	0.54
23	RCT filter 10	Cochrane B (2011) [1]<br>Dickersin et al. 2 (1994) [16]	0.82	0.26	0.54
24	RCT filter 4	Robinson and Dickersin 2 (2002) [13]	0.97	0.10	0.54
25	RCT filter 27	Jadad and McQuay (1993) [25]	0.80	0.27	0.54
26	RCT filter 5	Cochrane D (2011) [1]	0.97	0.10	0.54
27	RCT filter 2	Robinson and Dickersin 1 (2002) [13]	0.97	0.10	0.53
28	RCT filter 1	Duggan et al. (1997) [11]	0.99	0.04	0.51
29	RCT filter 35	Dumbrigue et al. 7 (2000) [12]	0.98	0.04	0.51
30	RCT filter 9	Glanville and Lefebvre strategy B (2006) [20]	0.83	0.16	0.50
31	RCT filter 8	Glanville and Lefebvre strategy A (2006) [20]	0.88	0.11	0.49
32	RCT filter 7	Glanville and Lefebvre strategy D (2006) [20]	0.84	0.13	0.49
33	RCT filter 30	Cochrane C (2011) [1]	0.74	0.09	0.42
		Dickersin et al. 3 (1994) [22]			
34	RCT filter 33	Dumbrigue et al. 2 (2000) [12]	0.41	0.36	0.39
35	RCT filter 34	Dumbrigue et al. 4 (2000) [12]	0.21	0.55	0.38
36	RCT filter 31	Dumbrigue et al. 5 (2000) [12]	0.59	0.13	0.36
37	RCT filter 36	Adams et al. standard (Adams 1) (1994) [17]	0.03	0.06	0.05
38	RCT filter 22	Glanville and Lefebvre strategy F (2006) [20]	0.02	0.07	0.04
		Dumbrigue et al. 8 (2000) [12]			

Note: Shaded rows are the Cochrane filters.
